# Shunt surgery in idiopathic normal pressure hydrocephalus is cost-effective—a cost utility analysis

**DOI:** 10.1007/s00701-017-3394-7

**Published:** 2017-11-17

**Authors:** Mats Tullberg, Josefine Persson, Jakob Petersen, Per Hellström, Carsten Wikkelsø, Åsa Lundgren-Nilsson

**Affiliations:** 1 Hydrocephalus Research Unit, Department of Clinical Neuroscience, Institute of Neuroscience and Physiology, Sahlgrenska Academy, University of Gothenburg, Sahlgrenska University Hospital, 41345 Gothenburg, Sweden; 2 Health Metrics, Sahlgrenska Academy, University of Gothenburg and Centre for Health Economics (CHEGU) at the University of Gothenburg, Gothenburg, Sweden

**Keywords:** Idiopathic normal pressure hydrocephalus, Shunt surgery, Cost effectiveness, Cost utility analysis, Quality of life, Markov model

## Abstract

**Background:**

The objective was to evaluate the cost-effectiveness of shunt surgery in patients with idiopathic normal pressure hydrocephalus (iNPH).

**Methods:**

Health-related quality of life was evaluated before and 6 months after surgery using the EQ-5D-3 L (EuroQOL group five-dimensions health survey) in 30 patients (median age, 71 years; range, 65–89 years) diagnosed with iNPH. The costs associated with shunt surgery were assessed by a detailed survey with interviews and extraction of register data concerning the cost of hospital care, primary care, residential care, home-care service and informal care. The cost of untreated patients was derived from the cost of dementia disorders in Sweden in 2012, as reported by the National Board of Health and Welfare. The cost effectiveness analysis used a decision-analytic Markov model. We used a societal perspective and a lifelong time horizon to estimate costs and effects. One-way sensitivity analysis and probabilistic sensitivity analysis were carried out to test the robustness of the model.

**Results:**

The shunt surgery model as the standard treatment in iNPH resulted in a gain of 2.2 life years and 1.7 quality-adjusted life years (QALY), along with an incremental cost per patient of €7,500/QALY. The sensitivity analysis showed that the results were not sensitive to changes in uncertain parameters or assumptions.

**Conclusions:**

Shunt surgery in iNPH, an underdiagnosed condition severely impairing elderly patients, is not only an effective medical treatment, it is also cost-effective, adding 2.2 additional life years and 1.7 QALYs at a low cost, a remarkable gain for an individual aged around 70 years.

## Introduction

Idiopathic normal pressure hydrocephalus (iNPH) is a treatable gait disorder and one of very few treatable causes of dementia, most often also causing balance and urinary disturbances [25]. Treatment by shunt surgery is effective with substantial clinical improvement in up to 80% of the patients [4, 25, 43]. Health-related quality of life (HRQoL) is lower in iNPH patients than in age-matched healthy individuals [19, 31], but improves in 86% of patients after surgery to almost the same level as in the normal population [31].

The prevalence of iNPH is high. Between 0.5 and 2.9% of people older than 65 years of age suffer from iNPH [5, 18, 40]. The disorder is underdiagnosed as well as undertreated; possibly only about 20% of affected patients undergo shunt surgery [6, 40, 42].

Taken together with improved diagnostic methods and increased knowledge about the disorder, the number of patients in need of shunt surgery for iNPH will most likely increase. This will challenge the allocation of healthcare resources.

The cost benefit of shunt surgery in iNPH has been addressed in only a few studies. In a retrospective study of patients with hydrocephalus, the Medicare expenditure for treated patients was lower than for untreated patients [41]. Based on data from the literature, the average 65-year-old patient receiving a shunt would gain an additional 1.7 QALYs [36]. The caregiver burden (Zarit burden interview score) also decreases after a shunt operation [22]. Kameda et al. [20] recently reported that shunt surgery yields a positive return on investment within less than 2 years.

The cost effectiveness of treating iNPH has not been investigated in a prospective study with patient experienced QoL and cost data, and the aim of the present study was to investigate this from a societal perspective, using a decision-analytic Markov model adapted to Swedish circumstances.

## Methods

### Patients

Thirty-seven patients (23 men, 14 women; median age, 70 years; range, 50–89 years), consecutively diagnosed with probable iNPH according to the American-European guidelines [34] at the Hydrocephalus Research Unit, were primarily included. Diagnosis was based on detailed assessment of typical clinical symptoms and signs and magnetic resonance imaging (MRI) findings according to the guidelines: no supplementary tests were used for inclusion. A lumbar puncture was performed in all cases, and the intracranial pressure (ICP) was determined (<18 mm Hg in all). The patients and their HRQoL data have been described in detail by Petersen et al. [31]. Seven patients aged <65 years were excluded and data for the remaining 30 retired patients aged ≥65 years were used in the cost-effectiveness analysis (16 men, 14 women; median age, 71; range, 65–89 years). Prior to surgery, the median Mini-Mental State Examination (MMSE) was 24 (range, 15–30), four patients had municipal home help service (cooking, cleaning, purchases, etc.), one was living in residential care, five patients needed help with personal activities of daily living (ADL), and 20 patients needed help with instrumental ADL, as measured by the Functional Independence Measure (FIM) and the Assessment of Motor and Process Skills (AMPS) instruments. All patients had their symptoms and signs scored on the iNPH scale [15] by an experienced neurologist, physiotherapist and neuropsychologist before and at re-evaluation 6 months after surgery. A change in the score (after surgery) of ≥5 points was categorised as improvement and <5 points as a poor outcome.

All patients received a ventriculo-peritoneal shunt with a Codman Hakim programmable valve and an anti-siphon device. All shunts were working at the 6-month postoperative evaluation.

### Analysis of cost effectiveness

#### The Markov model

**Fig. 1:**
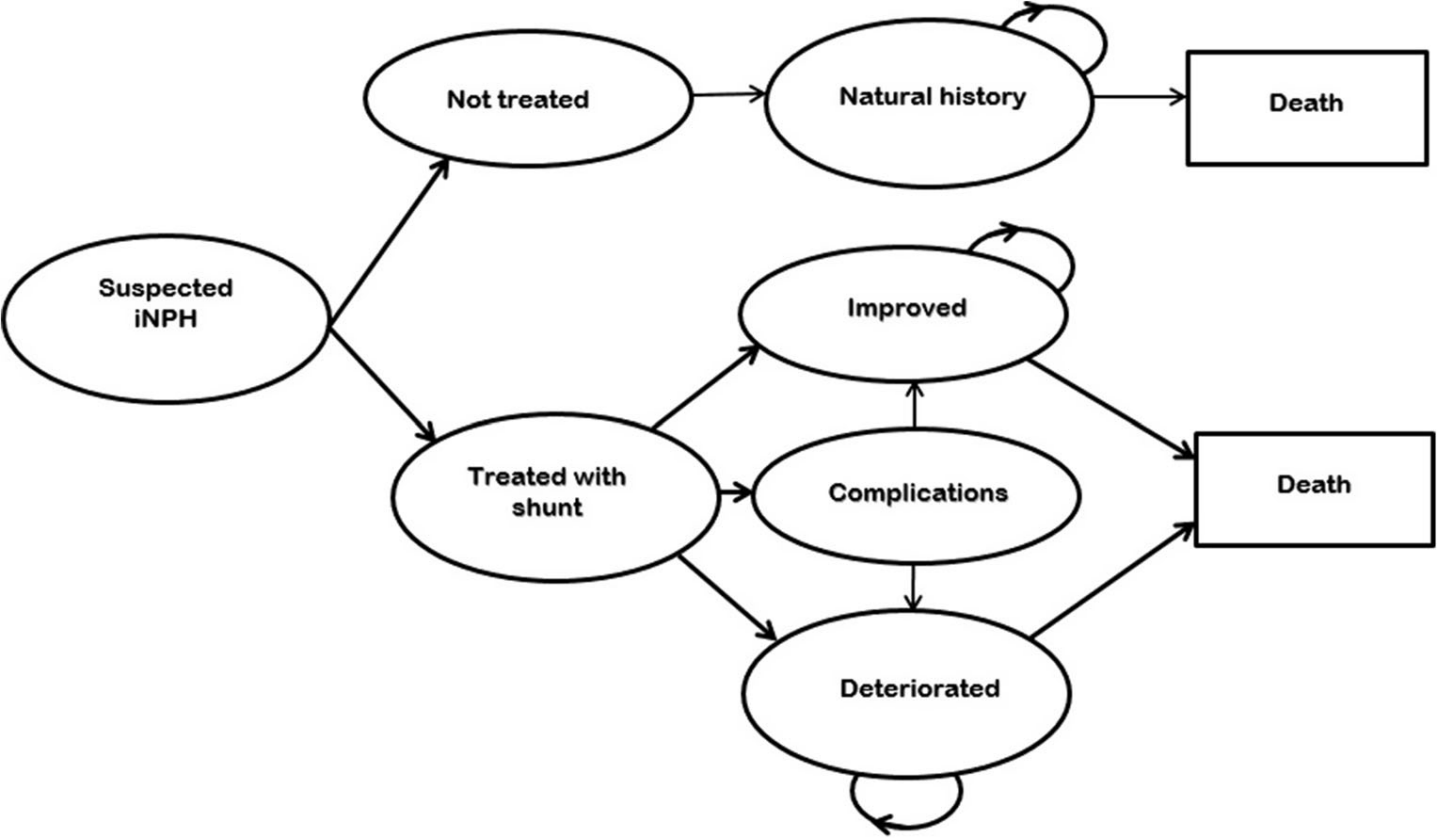
Transition probabilities for patients with shunt treatment or no treatment (natural course)

The cost effectiveness of shunt surgery in iNPH was analysed using a decision-analytic Markov model adapted to a Swedish setting. The Markov model is a mathematical model consisting of different health states that the simulated population can be in and move between [7]. The model was constructed with four health states for the shunt surgery pathway: “Improved”, “Complication”, “Deteriorated” and “Death” (death from iNPH or other causes). For the natural history, the model was constructed with two health states: “Natural history” and “Death” (death from iNPH or other causes) (Fig. 1). The input parameters in the model are presented in Table 1. The model simulated the course of events in a hypothetical cohort of 1,000 patients aged 70 years. The simulation model allowed us to estimate the lifelong costs and effects on HRQoL of performing shunt surgery in hypothetical individuals with iNPH and compare these with individuals who receive no treatment (natural history). The primary outcome of the model was the cost per gained quality-adjusted life year (QALY). QALY combines life years and HRQoL using a single index, often called QALY-weight, between 0 and 1, where 0 equals death and 1 represents perfect health. Thus, 1 QALY corresponds to 1 year of “perfect” health. The analysis had a societal perspective (including both the healthcare cost of hospital care, primary care and residential care and the cost of home-care service and care and support given by relatives). The long-term mortality risk (>1 year) for the state “Improved” was approximately 2.5 times [26, 41] higher than among the general population in Sweden [38]. The same applies to mortality for the states “Deteriorated” and “Natural history” but with an additional 10% risk of mortality [2]. A 3% discount rate was used for costs and effects throughout the model. Both deterministic and probabilistic sensitivity analyses were carried out to study the uncertainty of parameters and assumptions. The model was programmed in Microsoft Excel (Microsoft, Redmond, WA, USA).

**Table 1:** Parameter values in the Markov model with associated literature references where appropriate. Probabilities of support after surgery and costs associated with shunt surgery are based on data assessed in this study

Parameter	Value	Statistics/range^a^	Distribution	Reference
Probabilities first year				
Being improved	0.73		Beta	[43]
Complication	0.15		Beta	[11]
Being deteriorated	0.10		Beta	[25]
Probabilities after first year				
Complication	0.02		Beta	[11]
Being improved after complication	0.70		Beta	[2]
Being deteriorated	0.10		Beta	[25]
Annual mortality rate, deteriorated	0.10		Beta	[2]
Probabilities of support after surgery				
Living at residential care	0.07 (2 out of 30)		Uniform	
Home service	0.23 (7 out of 30)		Uniform	
Informal support	0.60 (18 out of 30)		Uniform	
Probabilities of support, natural history			Uniform	
Living in residential care	0.42		Uniform	[35]
Day care	0.06		Uniform	[35]
Cost (€)				
Surgery	10,200	8,160–12,240	Gamma	
Inpatient care	3,900	3,120–4,680	Gamma	
Outpatient care	1,700	1,360–2,040	Gamma	
Living in residential care	60,600	48,480–72,720	Gamma	[35]
Home service, after surgery	5,200	4,160–6,240	Gamma	[35]
Home service, natural history	6,900			
Day care, natural history	9,900		Gamma	[35]
Informal support, after surgery	6,600	5,280–7,920	Gamma	
Informal support, natural history	10,500			[35]
QALY-weights (mean)				
Improved	0.71	0.57–0.85	Beta	[31]
Complication, deteriorated, natural history	0.57	0.46–0.68	Beta	[31]
Discount rate (%)	3	0–5		

Costs are presented as annual cost per patient

^a^Range ± 20%

#### QALY-weights

Age-dependent QALY-weights based on the Swedish population were used in the model [9]; hence, the decline in HRQoL for reasons other than iNPH were also considered in the model. The QALY-weights were assessed with the EQ-5D-3 L (EuroQoL group five-dimensions health survey with three levels) [45], which is a generic preference-based measure of health, measured before and 6 months after surgery [31]. The value is based on data generated from a national general public survey using the time-trade-off (TTO) method to elicit mean TTO values at each health state [9]. Since there is no Swedish TTO tariff for the EQ-5D health states, the UK EQ-5D index tariff was used [39].

#### Costs

A detailed survey was made to assess the cost of the shunt-operated patients 6 months after shunt surgery, used as a source for costs in the Markov model. In this survey, all contacts with the health service and the associated costs were extracted from medical records. All patients were examined and prospectively interviewed personally by an occupational therapist (J.B.), before (at hospital) and 6 months after surgery (at hospital or in their homes). The number of hours per week that the relatives provided practical support and surveillance during their leisure time and the number of hours that the patients had home-care service were recorded and the corresponding costs estimated. The following costs associated with shunt surgery were assessed: (1) cost of healthcare, including hospital inpatient and outpatient visits, shunt surgery, primary care physician visits, contacts with a physiotherapist, occupational therapist or nurse; (2) cost of residential care in nursing home and home-help service costs (formal care); (3) cost of care given by relatives in their spare time (informal care). The annual cost was estimated by an extrapolation of data from the first 6 months shown in Table 1.

After shunt surgery, 7% (2 out of 30) of the patients in this survey lived in residential care, 23% (7 out of 30) received home care and 60% (18 out of 30) received informal support (Table 1). Formal and informal care were valued according to the valuation in the National Board of Health and Welfare report [35]. One hour of home help service was valued at €38 (SEK 378) and the annual cost of living in a nursing home was valued at €62,000 (SEK 606,000). The informal care was valued according to the opportunity cost method [10], where the informal care is valued as the person’s best alternative use of time. Loss of production was valued using the human capital approach [13], assuming that production loss can be valued at market price; i.e. gross salaries and payroll taxes. The opportunity cost of lost leisure time was estimated at 35% of the hourly loss of production of employed relatives [32]. One hour of informal care was valued at €15 (SEK 152), which is weighted to include both the loss of productivity and the loss of leisure time. The cost of the patient’s loss of production was not included, as the data applied to retired patients.

The cost of untreated patients with iNPH (natural history) was derived from the cost of dementia disorders in Sweden in 2012, as reported by the National Board of Health and Welfare [35]. According to this report, 42% lived in residential care and 58% lived in their own home, of whom 6% received day care. For those who lived on their own, it was estimated that the patients received 0.5 h of home care and 1.9 h of informal care per day.

All costs were adjusted according to the inflation rate for prices in 2016 and the exchange rates applicable on 3 November 2016 (Euro from Swedish Kronor and British Pound Sterling).

#### Sensitivity analysis

**Fig. 2:**
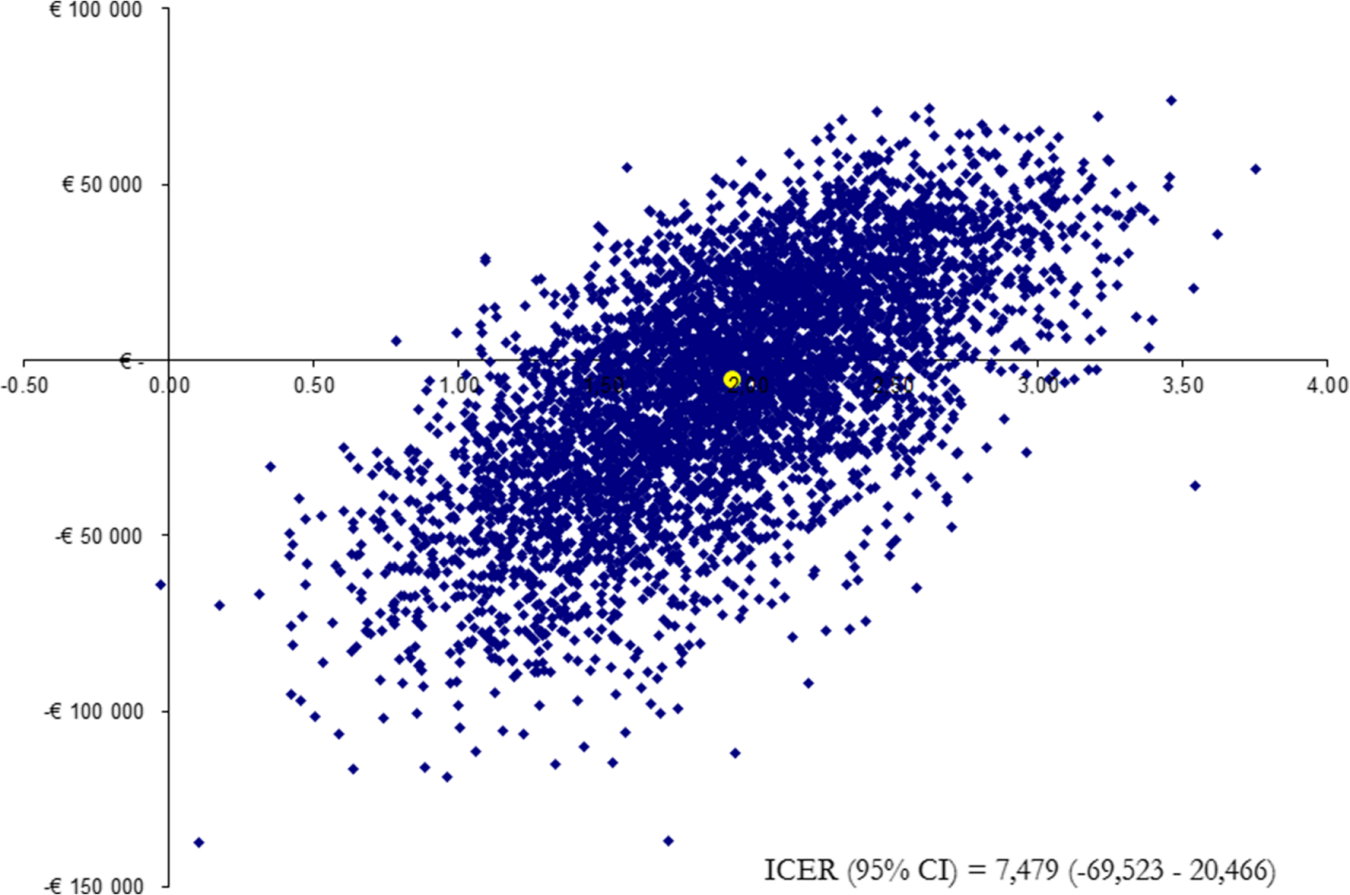
Incremental cost effectiveness plane

A one-way sensitivity analysis was conducted to evaluate the importance of uncertain parameters and assumptions (i.e. the risk of mortality, cost of municipal residential care and informal support in the cohort with natural history, utility weight of “improved” and “deteriorated”, the discount rate and with a healthcare perspective). A second-order probabilistic sensitivity analysis of the statistical uncertainty of parameters was undertaken using Monte Carlo simulation [8]. These parameters included the probabilities of state transitions, costs and QALYs for 5,000 bootstrap replicates. Since the costs are skewed, gamma distribution was used for the costs in the model. The result was plotted on an incremental cost effectiveness plane (Fig. 2). The willingness-to-pay (WTP) level was set based on current NICE guidelines [30] to £20,000/QALY (€22,150/QALY) for the lower threshold and £30,000/QALY (€33,220/QALY) for the upper threshold.

The Wilcoxon signed rank test was used to analyse changes in the iNPH scale score and HRQoL after shunt surgery.

The study was approved by the regional ethics committee in Gothenburg, Sweden. Written informed consent was obtained from the patients and/or their relatives.

## Results

**Fig. 3:**
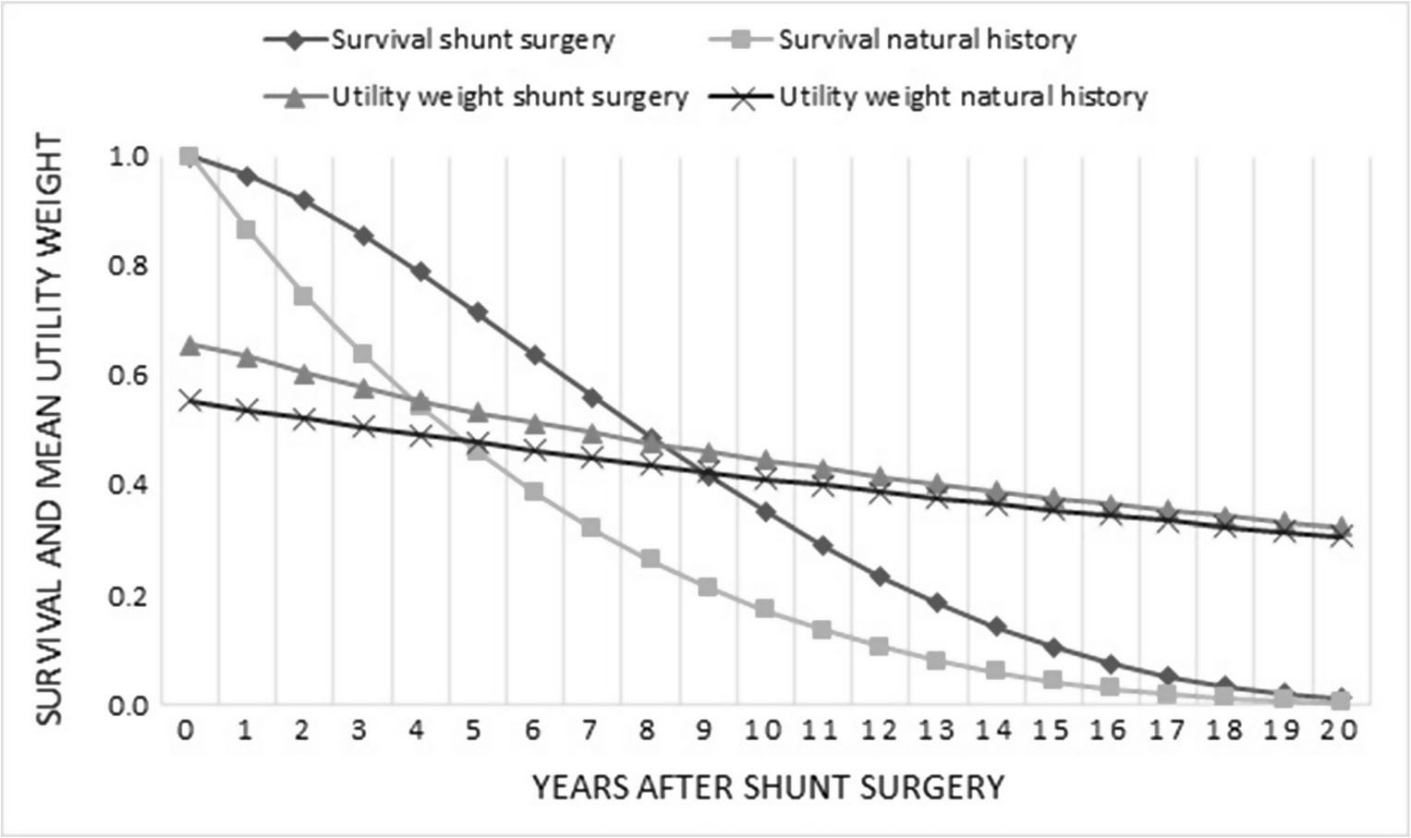
Monte Carlo simulation distribution plot illustrating the incremental cost effectiveness of shunt surgery compared with no treatment in iNPH

The lifelong cost-effectiveness model showed that shunt surgery resulted in a gain in both life years saved and QALYs of the simulated cohort, compared with natural history patients (Fig. 3). According to the model, shunt surgery resulted in a gain of 2.2 life years and 1.7 QALYs at an incremental cost of €13,000, compared with natural history. The incremental cost effectiveness ratio (ICER) was €7,500/QALY (Table 2). The incremental cost per life year saved was €6,000.

**Table 2:** Results of the cost effectiveness analysis with shunt surgery associated incremental gain in life years and QALYs and the ICER of shunt surgery

Option	Cost (€)	Incremental cost (€)	Life years	Incremental life years	QALY	Incremental QALY	ICER
Shunt	173,000		6.60		4.25		
Natural history	160,000	13,000	4.42	2.17	2.52	1.73	7,500

*QALY* quality adjusted life years, *ICER* incremental cost effectiveness ratio

The estimated annual costs after surgery and for the natural history patients are shown in Table 1.

### Sensitivity analysis

The one-way sensitivity analysis showed that the results were not sensitive to major changes in risk of mortality, cost of municipal residential care and cost of informal care for the cohort with the natural history, utility weight for the state “Improved” and “Deteriorated” as well as the discount rate (Table 3). Shunt surgery still generated a low cost per gained QALY. With a healthcare perspective, the cost would be €4,300 per gained QALY. The reliability of the results was also tested with probabilistic analyses. The Monte Carlo simulation exercise indicates robustness of the model: shunt surgery is cost-effective with a probability of 98.5% (lower WTP threshold) and 100% (upper WTP threshold) of the Monte Carlo simulations (Fig. 2).

**Table 3:** Results of the one-way sensitivity analysis

Alternative	Incremental Cost (€)	Incremental QALY	ICER
Risk of mortality			
8%	900	1.57	580
12%	23,000	1.87	12,300
Cost of municipal residential care			
€48,480 (−20%)	14,200	1.73	8,200
€72,720 (+20%)	11,800	1.73	6,800
Cost of informal support, natural history			
€8,000 (−20%)	14,700	1.73	8,500
€12,600 (+20%)	11,300	1.73	6,500
QALY improved			
0.57 (−20%)	13,000	1.24	10,500
0.85 (+20%)	13,000	2.23	5,800
QALY deteriorated			
0.45 (−20%)	13,000	1.90	6,800
0.69 (+20%)	13,000	1,57	8,300
Healthcare perspective	7,400	1.73	4,300
Discount rate			
0%	23,700	2.10	11,300
5%	7,900	1.54	5,200

*QALY* quality adjusted life years, *ICER* incremental cost effectiveness ratio

### Patient outcomes

Six months after shunt surgery, 20 (67%) of the patients showed improvement on the iNPH scale (*p* < 0.001). The iNPH scale score increased from 54.9 (45.6–64.3) [median, interquartile range (IQR)] preoperatively to 71.1 (59.9–79.5) postoperatively with a change of 15.0 (1.6–22.4). One patient could not be properly evaluated, and this patient’s condition was considered not improved. HRQoL was improved in 24 (83%) of 29 patients 6 months after surgery (*p* < 0.001). EQ-5D visual analogue scale increased from 55 (44.5–70) [median score (IQR)] preoperatively to 80 (58.75–85) postoperatively. Seven patients (23%) suffered from complications during the first 6 months: three patients had subdural haematomas that could be treated by temporarily increasing the shunt opening pressure; in four cases, surgical revision was required (for shunt dysfunction in three cases and infection in one case).

## Discussion

This study shows that shunt surgery is an inexpensive and cost-effective treatment in iNPH. The average iNPH patient gains 2.2 life years and 1.7 QALY, a substantial gain for a patient aged around 70 years. In comparison, Donezepil treatment in Alzheimer’s disease gives an estimated gain of 0.11 QALYs at a considerably higher incremental cost effectiveness ratio (ICER) [12], whereas endovascular thrombectomy in acute stroke adds 0.4 life years and 0.99 QALY [3].

The ICER for shunt surgery was €7,500/QALY and the cost per life year saved was €6,000. Even though we used a societal perspective and included other costs than healthcare costs, it is well below the UK National Institute for Health and Care Excellence (NICE) nominal healthcare cost-per-QALY threshold for new interventions. Thus, shunt surgery would most probably have been strongly recommended if introduced as a new intervention. Compared to other established surgical procedures, shunt surgery has a low extra cost per QALY gained, matching, for example, hip replacement surgery (Table 4). Our results corroborate with a recent report by Kameda et al. [20] who also concluded that shunt surgery is cost effective, even though they found a higher cost/QALY (US$30,000–41,000/QALY equalling €28,000–39,000/QALY), probably due to a different study design with indirect estimations of costs and utility values.

**Table 4:** Extra cost per QALY gained for different established surgical procedures

Surgical procedure	€/QALY	
Hip replacement, 1 year	6,750	[33]
Shunt surgery for iNPH	7,500	
Knee replacement, 1 year	14,000	[33]
PCI for MI	17,000	[44]
DBS for Parkinson’s disease	23,100	[21]
Intractable partial epilepsy	24,500	[24]

*PCI* percutaneous coronary intervention, *MI* myocardial infarction, *DBS* deep brain stimulation, *QALY* quality adjusted life years

This study was performed in a Swedish setting. We are, however, convinced that the results reported here are valid in developed countries throughout the world. The surgical procedure and the effects of shunting are comparable in most specialised centres. Also, costs for the “Deteriorated” state used here are within the range of the societal costs presented for other countries. The societal cost per person with dementia in Europe was estimated to US$24,565 (€22,168) and in North America to US$36,603 (€33,301) in 2009 years prices [46]. A recent study from Sweden, estimated the annual cost per person with dementia to €43,259 [1]. The yearly monetary cost in the United States per person with dementia was estimated to US$56,290 (€50,800) or US$41,689 (€37,620), depending on which method was used to value informal care [17].

The patients in our study were consecutively included, presented typical clinical features, and 67% were improved on the iNPH scale, a slightly lower figure than in many contemporary studies [43]. However, 83% reported improved HRQOL. The overall complication rate of 23% was somewhat high, but the revision rate of 13% is comparable with earlier studies [11]. All patients were diagnosed based on typical clinical symptoms and MRI findings according to international guidelines [34]. No supplementary tests, such as a cerebrospinal fluid (CSF) drainage test or lumbar infusion test, were used for inclusion. Inclusion of supplementary tests for the selection of patients for surgery has been discussed and recommended in several recent papers [14, 23, 27–29]. These tests are widely used due to their ability to identify shunt responders and adding these tests probably increase response rate to shunt surgery. The lower success rate in this study could be an effect of not using these tests. However, the European multicentre study [25] showed that when no supplementary tests are used, the number of patients improved by shunt surgery is 82%, which is similar to studies using such tests for inclusion. The dilemma of using or not using supplementary tests is further strengthened by the fact that iNPH patients that would benefit from surgery would be excluded by the use of supplementary tests as selection criteria [25]. Altogether, we consider the patient sample to be representative and the reported results valid. It was also established that the shunts worked in all the participants.

The input parameters used in the model were derived from the recent literature. Results from the European multicentre iNPH study [25], a large prospective longitudinal study with results comparable with those of many previous reports, and a recent systematic review of the outcome of shunt surgery in iNPH [20] formed the basis for the calculation of model outcome probabilities. A 73% probability of being improved after shunt surgery was used, corresponding to outcomes after more than 3 years reported in studies since 2006 [20], a figure that also corresponds to our experience and previous reports from our centre. Even if this figure is higher than recently reported in a large quality registry based study [37], we believe it is reasonable. For complication probabilities, data from a previous study at our centre, showing a rate of 13% for complications in need of intervention during the first year and 2% thereafter, were used [11]. These figures are comparable with other reports [43] and higher than in a recent report on cost-effectiveness [20] why we do not consider the complication rate underestimated. The mortality for the improved state was estimated according to the age-adjusted mortality rate for the normal Swedish population with an increase of 2.5 times. The same applies to the mortality for the deteriorated state, but with an additional 10% risk of mortality. We consider these input parameters to be the best possible estimates available. Also, the sensitivity analysis showed the robustness of the model—the results were not sensitive to major changes in long-term costs or utility weights—and, although some of the input parameters may be slightly uncertain, the main results will remain, even if the input parameters are varied.

The annual cost for the state “Deteriorated” was derived from the report by the National Board of Health and Welfare [35], where 78% of the cost is related to home care and the cost of living in a nursing home. According to the report, 42% of patients with dementia live in nursing homes, which is higher than the rate reported here for patients with iNPH. Shunt surgery will reduce patients’ need for assisted living; furthermore, in our experience, arranging accommodation in a residential home is often neglected for patients with suspected iNPH at the time of diagnosis, probably due to poor knowledge of the disorder and its prognosis. The natural course of iNPH is sparsely studied, with few reports in the literature, and, to our knowledge, there are no reports of the care burden and the cost of untreated patients. Also, for ethical reasons, a prospective study of the natural course would hardly be acceptable, considering the effective treatment available. We consider the reported cost of dementia disorders in Sweden a good estimate of the cost of untreated iNPH patients.

No supplementary tests, such as the CSF tap-test or lumbar infusion test, were used when including patients in this study. Costs for these tests are very low compared to the surgical procedure and are probably compensated by the extensive clinical testing performed on all of our patients. Again, sensitivity analysis showed that the results were not sensitive to major changes in parameters. We consider the results reported here valid also for centres using supplementary tests.

We used a societal perspective in the analysis and included the cost of informal care provided by relatives. However, we did not include the caregivers’ utility weight in the model. A previous review has shown that self-reported health was strongly related to the time spent caring and to the carer’s perceived burden [16]. Thus, since the cost of informal care is similar for the states “Improved” and “Deteriorated”, we assume that the burden for the caregivers is equal.

In general, simulation models have limitations when estimating cost effectiveness, often due to the lack of long-term data, resulting in major uncertainties of the long-term effects of the treatment in terms of costs, HRQoL and mortality. One weakness in our study is the small sample, mainly caused by the difficulty of undertaking such a detailed survey with personal interviews in larger samples. We chose to exclude seven patients aged <65 years and included only retired patients ≥65 years in order to get a patient group most representative of the target population. As stated earlier, we believe that the sample is representative, but further studies with larger samples are warranted. For this reason, results from other published studies have been included in the model, as probabilities of moving between the health states and the annual mortality rate. The uncertainties should be analysed with sensitivity analyses, as in this study.

One strength of the study is the rigorous recording of related costs after shunt surgery. All examinations were performed by a skilled occupational therapist (J.B.), either in connection with patient visits to the hospital or in the patient’s home. Relatives were always interviewed. Costs were also estimated through a meticulous evaluation of medical records. We believe that this objective procedure, evaluating qualitative and quantitative aspects of care, yields the most correct data possible. Further, HRQoL was measured using a reliable and widely used instrument.

## Conclusions

This study shows that shunt surgery**,** an effective medical treatment for iNPH, is cost-effective and could therefore be recommended; shunt surgery as a standard treatment resulted in a gain of 2.2 life years and 1.7 quality-adjusted life years, along with an incremental cost per patient of €7,500/QALY. This is a substantial gain for elderly persons and a greater gain and lower cost/QALY than for many comparable standard interventions. In light of the increasing awareness of iNPH, we believe that the results reported here are highly relevant to the future allocation of healthcare resources.
